# High Engraftment and Metastatic Rates in Orthotopic Xenograft Models of Gastric Cancer via Direct Implantation of Tumor Cell Suspensions

**DOI:** 10.3390/cancers16040759

**Published:** 2024-02-12

**Authors:** Chao Wang, Guo-Min Xie, Li-Ping Zhang, Shuo Yan, Jia-Li Xu, Yun-Lin Han, Ming-Jie Luo, Jia-Nan Gong

**Affiliations:** 1 National Human Diseases Animal Model Resource Center, National Center of Technology Innovation for Animal Model, NHC Key Laboratory of Human Disease Comparative Medicine, The Institute of Laboratory Animal Sciences, Chinese Academy of Medical Sciences & Peking Union Medical College, Beijing 100021, China; wangchao@cnilas.org (C.W.); xieguomin@cnilas.org (G.-M.X.); zhangliping@cnilas.org (L.-P.Z.); yanshuo@cnilas.org (S.Y.); xujiali@pumc.edu.cn (J.-L.X.); 18510165683@163.com (Y.-L.H.); 2 State Key Laboratory of Ophthalmology, Zhongshan Ophthalmic Center, Sun Yat-Sen University, Guangzhou 510080, China; zoc_mjluo@yeah.net

**Keywords:** gastric cancer, orthotopic xenograft model, tumor invasion, metastasis

## Abstract

**Simple Summary:**

Despite remarkable progress in treating early-stage gastric cancer (GC), the clinical outcomes for patients with advanced disease remain very poor. Tissue invasion and metastasis constitute the major causes of cancer-related deaths, including GC. This highlights the urgent need to develop animal models that can recapitulate these processes to develop novel therapeutic strategies. We developed a highly reproducible and cost-effective procedure to establish orthotopic GC xenografts showing high engraftment and metastatic rates via the direct implantation of tumor cell suspensions. Compared with the routine method to establish orthotopic xenograft models by engrafting intact tumor fragments, our approach significantly shortens the experimental timeline and allows for the flexible adjustment of the number of tumor cells implanted to control the rate of tumor progression. Both dose- and time-dependent progressions of tumor invasion and metastasis were nicely recapitulated in our model. Our work provides valuable tools for studying GC progression and metastasis and developing effective therapies.

**Abstract:**

Although the implantation of intact tumor fragments is a common practice to generate orthotopic xenografts to study tumor invasion and metastasis, the direct implantation of tumor cell suspensions is necessary when prior manipulations of tumor cells are required. However, the establishment of orthotopic xenografts using tumor cell suspensions is not mature, and a comparative study directly comparing their engraftment and metastatic capabilities is lacking. It is unclear whether tumor fragments are superior to cell suspensions for successful engraftment and metastasis. In this study, we employed three GC cell lines with varying metastatic capacities to stably express firefly luciferase for monitoring tumor progression in real time. We successfully minimized the risk of cell leakage during the orthotopic injection of tumor cell suspensions without Corning Matrigel by systematically optimizing the surgical procedure, injection volume, and needle size options. Comparable high engraftment and metastatic rates between these two methods were demonstrated using MKN-45 cells with a strong metastatic ability. Importantly, our approach can adjust the rate of tumor progression flexibly and cuts the experimental timeline from 10–12 weeks (for tumor fragments) to 4–5 weeks. Collectively, we provided a highly reproducible procedure with a shortened experimental timeline and low cost for establishing orthotopic GC xenografts via the direct implantation of tumor cell suspensions.

## 1. Introduction

Gastric cancer (GC) is one of the leading causes of cancer-related deaths worldwide. The incidence and mortality of gastric cancer are ranked fifth and fourth, respectively, among all the cancer types according to GLOBOCAN 2020 [1]. Tissue invasion and seeding at distant sites to form metastasis constitute the major causes of most cancer-related deaths, including GC [2,3,4]. Animal models play a critical role in understanding the biological processes of cancer development and in the pre-clinical evaluation of potential therapeutic targets or strategies. Therefore, the development of animal models that can replicate these processes provides important tools for studying GC pathogenesis and for the pre-clinical testing of potential therapeutic interventions.

Orthotopic xenograft models are widely used for studying tumor invasion and metastasis [5,6,7,8]. The implantation of intact tumor fragments is commonly used to generate orthotopic xenograft models for both patient-derived primary samples and cancer cell lines [9,10,11]. In recent years, the orthotopic implantation of tumor cell suspensions using different techniques has also been reported and has shown various engraftment and metastatic rates [12,13,14]. Limitations, such as low engraftment and metastatic efficiencies [15] as well as the high risk of leakage, compromise the practical application of the orthotopic implantation of tumor cell suspensions. Nevertheless, the orthotopic engraftment of tumor cell suspensions is particularly valuable when primary samples or cancer cell lines require prior manipulations, such as ex vivo culturing, drug treatments, gene editing, or genetic screening. However, the methodology to establish orthotopic xenografts using tumor cell suspensions has not been well established. A comparative study directly comparing engraftment and metastatic capabilities is lacking. Therefore, there is an unmet need to develop a highly reproducible procedure that ensures a high success rate for generating orthotopic models using tumor cells in suspensions.

In this study, we employed three GC cell lines with varying metastatic capacities to stably express luciferase for monitoring in situ tumor growth, metastasis, or cell leakage in real time. We successfully minimized the risk of cell leakage during the orthotopic injection of tumor cells in PBS without Matrigel by systematically optimizing the surgical procedure, injection volume (≤40 μL), and needle size options (29 G or 30 G). Minimal risk of cell leakage via our procedure was confirmed in both HGC-27 and 23132/87 orthotopic xenograft models, which showed no evidence of metastasis by week 4 and week 5, respectively. Using highly metastatic MKN-45 cells, we demonstrated comparable high engraftment and metastatic rates for engrafting intact tumor fragments and tumor cell suspensions. Importantly, our approach allows for the flexible adjustment of the number of tumor cells implanted to control the rate of tumor progression and significantly shortens the experimental timeline to 4–5 weeks from 10–12 weeks for engrafting tumor fragments, which is advantageous for conducting drug screenings and evaluating therapeutic interventions. These models serve as valuable tools for elucidating the biological processes of GC tumor progression and metastasis and for evaluating potential antitumor or anti-metastatic therapies.

## 2. Materials and Methods

### 2.1. Mice

BALB/c nude mice (female, 5–6 weeks old) were purchased from Sibeifu Biotechnology Co., Ltd. (Beijing, China) and maintained in a specific-pathogen-free (SPF) animal research facility. Upon arrival, the mice were allowed to acclimate for 2–3 days before the experiment. The study was approved by the Institutional Animal Care and Use Committee of the Institute of Laboratory Animal Science and the Peking Union Medical College (IACUC 21001).

### 2.2. Cell Culture

Human GC cell lines MKN-45, 23132/87, and HGC-27 were purchased from Cobioer (Nanjing, China). All the cell lines were cultured in RPMI-1640 medium (Gibco, Oakland, CA, USA) supplemented with 10% fetal bovine serum (Gibco, Oakland, CA, USA) at 37 °C with 5% CO_2_. The regular authentication of the cell lines was performed using STR (short tandem repeat) profiling at RuiBiotech (Beijing, China). The cells were tested quarterly using the MycoAlert mycoplasma detection kit (Beyotime, Nanjing, China) and were consistently negative for mycoplasma.

### 2.3. Production of Lentiviral Particles and Transduction of Cells

The lentivirus packaging plasmids pMDLg/pRRE [16], pRSV-Rev [16], and pCMV VSV-G [17] were transiently transfected into HEK293T cells with the lentiviral construct of firefly luciferase (plenti-SFFV-luciferase-puro vector, genecarer, Xi’an, China) using the Lipofectamine™ 3000 transfection reagent (Thermo, Waltham, MA, USA). A supernatant containing infectious virus particles was harvested 48 h later. A second viral harvest was made following a further 24 h of incubation with fresh medium. The supernatant containing the viruses was filtered through a 0.45 μm filter and stored at 4 °C for use or at −80 °C until used.

GC cells were seeded into 6-well plates at 5 × 10^5^ cells/well. An equivalent volume of the virus-containing culture medium was added along with polybrene (Sigma, St. Louis, MO, USA) to a final concentration of 5 μg/mL. The cells were spin infected (1800× *g*, 25 °C, 1 h) and incubated at 37 °C for 24 h. The cells were then washed and re-cultured in fresh medium, as described above, for 3–4 days. On day 5, the medium was replaced with a selection medium containing the optimal dose of puromycin (1 μg/mL), as pre-determined in a cell viability assay. The cells were maintained in the selection medium for 5–7 days. At least 50% of the infection efficiency was achieved before the selection, and luciferase positivity was confirmed using a multi-mode microplate reader (Perkin Elmer EnSpire, Waltham, MA, USA).

### 2.4. Orthotopic Implantation of Tumor Fragments

To acquire intact tumor fragments for the orthotopic implantation, 5 × 10^6^ luciferase-expressing MKN-45 cells were injected subcutaneously into the flanks of 5–6-week-old female BALB/c nude mice. Tumors were collected 2–3 weeks later when they reached approximately 1 cm^3^, and the macroscopically viable tumor tissue avoiding the central necrotic area of the tumors was minced into small (~1 mm^3^) fragments for the subsequent orthotopic implantation.

To establish the orthotopic xenograft model, 5–6-week-old female BALB/c nude mice were anesthetized via an intraperitoneal (i.p.) injection of ketamine (100 mg/kg bodyweight), and the left side of the mouse abdomen was sterilized with alcohol, after which a 5–10 mm incision was made into the skin and peritoneum. The stomach was then exteriorized using surgical forceps, and a 1–2 mm incision was made into the serosa of the gastric corpus, after which microsurgical scissors were used to widen the entry to the subserosal layer to obtain one small tissue pocket. One donor tumor fragment was subsequently placed into the tissue pocket using microsurgical forceps. The entry was then fixed with a piece of medical gelatin sponge soaked with a tissue adhesive. The stomach was then relocated in the abdominal cavity, and the abdominal wall and skin were closed with 4–0 absorbable sutures.

### 2.5. Orthotopic Implantation of Tumor Cell Suspensions

To acquire the cell suspension for the orthotopic implantation, the luciferase-expressing MKN-45 cells in the culture were collected and re-suspended in phosphate-buffered saline (PBS). The cell density and viability were then measured using the Countess II automated cell counter (Thermo Fisher, Waltham, MA, USA), with a cell viability of >90% used for the subsequent injection.

For the orthotopic injection of the tumor cell suspensions, 5–6-week-old female BALB/c nude mice were anesthetized via an i.p. injection of ketamine (100 mg/kg bodyweight), and the left side of the mouse abdomen was sterilized with alcohol, after which a 5–10 mm incision was made into the skin and peritoneum. The stomach was then exteriorized using surgical forceps for the subsequent implantation. The entry site of the injection started from the greater curvature of the gastric corpus, avoiding blood vessels, with a needle extending 6–7 mm into the sub-serosal layer to the gastric corpus/antrum junction. The cell suspension was then injected slowly into the sub-serous layer of the gastric corpus/antrum junction. When the injection was completed, the needle was withdrawn slowly, with a bulge observed in the sub-serous area. The stomach was then relocated in the abdominal cavity, and the abdominal wall and skin were closed with 4–0 absorbable sutures.

### 2.6. In Vivo and Ex Vivo Bioluminescence Imaging

D-Luciferin (PerkinElmer, Waltham, MA, USA) was injected intraperitoneally at 150 mg/kg, and the mice were allowed to move freely to aid in the distribution of the luciferin. Ten minutes later, the mice were anesthetized using isoflurane and imaged using an In Vivo Imaging Systems (IVIS) Lumina II instrument (Perkin Elmer). The ex vivo imaging of the major tissues/organs was performed immediately after the in vivo imaging. Bioluminescence imaging was performed according to the manufacturer’s instructions, and the bioluminescence intensity was presented as the average radiance (photons/s/cm^2^/sr).

### 2.7. Tumor Volume Measurement

The greatest longitudinal diameter (length) and the greatest transverse diameter (width) were measured using a vernier caliper. Tumor volumes were calculated according to the formula 0.5 × length × width^2^.

### 2.8. H&E and Ki67 Stainings

The extracted mouse organs were fixed in 10% formalin for 24 h, embedded in paraffin after dehydration, cut into 4 μm sections, and then immobilized on glass slides, followed by decalcification. Hematoxylin and eosin (H&E) staining was performed according to the manufacturer’s instructions.

For immunohistochemistry staining, histologic sections were subjected to antigen recovery by microwave heating in a citrate buffer. The sections were then permeabilized using 0.5% Triton X-100 (Solarbio, Beijing, China) in PBS for 15 min. After blocking with goat serum at room temperature for 30 min, the fixed mouse organs were incubated with the primary antibody against Ki67 (Abcam, Waltham, MA, USA) at 4 °C overnight, followed by incubation with a horseradish-peroxidase-conjugated secondary antibody at room temperature for 30 min. The staining of the positive cells was finally detected using diaminobenzidine (DAB).

### 2.9. Statistical Analysis

GraphPad software (version 9) was used for the statistical analysis. All the data were expressed as the mean ± standard error of the mean (SEM) and analyzed using either a two-tailed unpaired Student’s *t*-test or one-way ANOVA, with *p*-values < 0.05 considered to be statistically significant.

## 3. Results

### 3.1. Establishment of MKN-45 Orthotopic Xenograft Model by Transplanting Subcutaneous Tumor Fragments from Donor Mice

**Figure 1:**
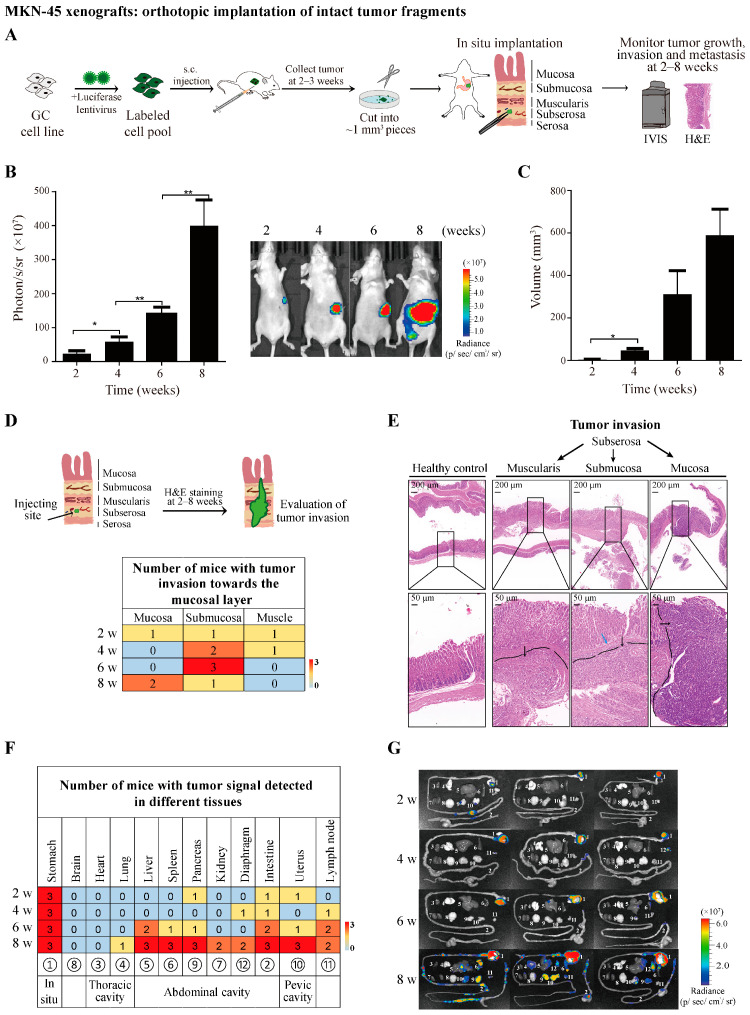
Establishment of MKN-45 orthotopic xenograft model by transplanting subcutaneous tumor fragments from donor mice. (**A**) Flowchart of the experiment. (**B**) In vivo monitoring of tumor growth and progression using bioluminescence imaging. Quantitative analysis of bioluminescence intensity (**left**) and representative images at 2, 4, 6, and 8 weeks (**right**) after tumor implantation. (**C**) Primary tumor volumes at 2, 4, 6, and 8 weeks after orthotopic transplantation. (**D**) Degree of tumor invasion determined by H&E staining across the transverse section of the gastric wall. Top: diagram depicting the transverse section of the gastric wall. From inside to outside: mucosa, submucosa, muscularis, subserosa, and serosa. Bottom: summary of number of mice with tumor invasion toward the mucosal layer. (**E**) Representative images of H&E staining in healthy stomach tissue and tumor invasion toward the muscular, submucosal, or mucosal layer. The black lines and arrows indicate the boundary and region of the tumor mass that invaded different layers of stomach tissue. The blue arrow indicates the breakthrough site of tumor cells from the layer of the submucosa to the mucosa. (**F**) Summary of tumor metastasis. Ex vivo imaging of major tissues and organs was performed immediately after in vivo imaging. Summary of number of mice with tumor signal detected in different tissues and organs. (**G**) Representative ex vivo bioluminescence images at 2, 4, 6, and 8 weeks after tumor implantation. The following tissues and organs are shown: 1. stomach, 2. intestine, 3. heart, 4. lung, 5. liver, 6. spleen, 7. kidney, 8. brain, 9. pancreas, 10. uterus, 11. lymph nodes from axilla and inguinal areas, and 12. diaphragm. Bioluminescence intensity in this study is presented as the average radiance (photons/s/cm^2^/sr). Data represent the mean ± SEM of 3 mice in each group. * *p* < 0.05; ** *p* < 0.01.

The implantation of intact tumor fragments is routinely used to generate orthotopic GC xenograft models with high engraftment and metastatic rates. Thus, before we performed the orthotopic transplantation of the tumor cell suspensions, we first established the orthotopic mouse model as the positive control by transplanting subcutaneous tumor fragments from donor mice to ensure high rates of tumor engraftment and metastasis. To do this, we selected a poorly differentiated cell line, MKN-45, and labeled the cells with a stable expression of firefly luciferase for monitoring tumor growth and progression using IVIS. The orthotopic model was constructed as most papers have reported [11,18,19,20,21,22]. In short, 5 × 10^6^ firefly-luciferase-expressing MKN-45 cells were first injected subcutaneously into the flanks of female BALB/c nude mice. The subcutaneous tumor was subsequently collected when it reached approximately 1 cm^3^ (in ~3 weeks) and cut into small fragments (~1 mm^3^). For the surgery for the orthotopic transplantation of tumor fragments, we first used microsurgical scissors to make an incision in the outermost layer of the gastric corpus (serosa) and then widened the entry to the space under the serosa to prepare one small tissue pocket to embed the tumor fragment. One donor tumor fragment was subsequently placed in the tissue pocket using microsurgical forceps. The entry was then fixed with a piece of medical gelatin sponge soaked with a tissue adhesive to avoid the tumor fragment slipping out (Figure 1A).

Tumor growth and progression were monitored every 2 weeks using an in vivo imaging system, and the quantification of the bioluminescence intensity showed time-dependent tumor progression after implantation (Figure 1B). Consistently, tumor volumes increased over time as well, with 100% (12/12) engraftment rates achieved (Figure 1C). To determine whether this model could mimic tumor invasion, H&E staining was conducted across the transverse section of the stomach to measure the degree of invasion toward the direction of the mucosa (Figure 1D,E). As early as 2 weeks after the orthotopic implantation, various degrees of invasion in the layer of the muscularis, submucosa, or mucosa were detected. A roughly time-dependent progression of tumor invasion was also observed (Figure 1D). To examine the tumor metastasis, every 2 weeks, mice were culled immediately after in vivo imaging, and major tissues/organs were then collected for ex vivo imaging. At 2 weeks after the orthotopic implantation of the tumor fragment, weak tumor signals were detected in the small intestine, pancreas, and uterus in 1 out of 3 mice. The metastatic rate increased over time, with massive tumor signals detected in multiple organs in all three mice at 8 weeks (Figure 1F,G). Our results confirmed the high engraftment and metastatic rates of the MKN-45 cells, which were thus used for the subsequent study, to set up the system for the orthotopic transplantation of the tumor cell suspensions.

### 3.2. Minimized Risk of Cell Leakage by Orthotopic Injection of Tumor Cell Suspensions in PBS through Optimized Injection Volume and Appropriate Needle Size

**Figure 2:**
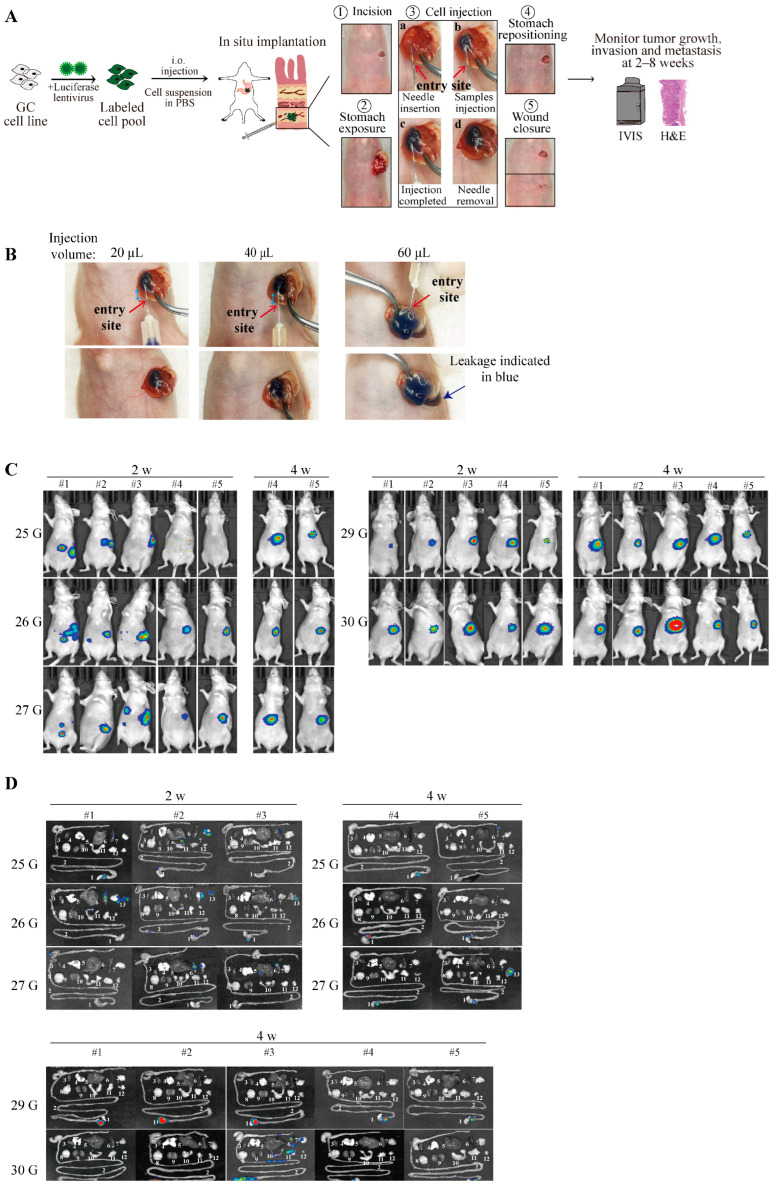
Optimization of injection volume and needle size for orthotopic injection of tumor cell suspensions. (**A**) Flowchart of the experiment. (**B**) Effect of different injection volumes on cell leakage. A total of 5 × 10^6^ MKN-45 cells suspended in 20 μL, 40 μL, or 60 μL of PBS with the addition of 1/5 the volume of trypan blue was inoculated in the sub-serosal area of the gastric corpus/antrum junction using a 1 mL syringe with a 30 G needle. Red arrows indicate the entry sites of injection. The distance between the entry site and the boundary of tumor cells after injection is highlighted by blue arrows. (**C**) Effect of different needle sizes on cell leakage. In vivo bioluminescence imaging at 2 and 4 weeks after orthotopic injection of 5 × 10^4^ luciferase-expressing MKN-45 cells suspended in 20 µL of PBS using 1 mL syringes with indicated diameters of needles. Five mice were used in each group. (**D**) Ex vivo bioluminescence imaging confirming the effect of different needle sizes on cell leakage. The following tissues and organs are shown: 1. stomach, 2. intestine, 3. heart, 4. lung, 5. liver, 6. spleen, 7. pancreas, 8. brain, 9. kidney, 10. uterus, 11. mesentery, 12. lymph nodes from axilla and inguinal areas, and 13. peritoneum. To better visualize the signal of the cell leakage, the diameter of the bioluminescence scale bar is set to ‘auto’ in (**C**,**D**).

Cell leakage is one of the major limitations that hamper the application of the orthotopic implantation of cell suspensions. Several factors, such as suboptimal surgical procedures and inappropriate injection volume or needle size, can increase the risk of cell leakage into the peritoneal cavity. Approaches, including mixing cells with Matrigel upon injection or using a cotton swab to press against the injection site for ≥20 s, have been reported in previous studies to reduce the risk of cell leakage [18,19]. A low injection volume is expected to reduce the risk of cell leakage. Although the orthotopic injection of 50 μL of the cell mixture has previously been reported [18], it is not indicated in most studies [19,23].During the process to optimize the surgical procedure, we found that the sample draw and injection of the cell–Matrigel mixture at a low volume (50 μL or less), especially with a high cell number, are hard to perform in practical operations owing to the stickiness and rapid solidification of the Matrigel. Therefore, we chose to use cells suspended in PBS for the orthotopic injection in the following experiments. The surgical procedure is summarized in Figure 2A. Briefly, the entry site of the injection starts from the greater curvature of the gastric corpus, avoiding blood vessels, with the needle extending 6–7 mm into the sub-serosal layer to the gastric corpus/antrum junction. The cell suspension was then injected slowly, with a bulge observed in the subserous area. It is crucial to maintain a sufficient distance between the entry site and loading site (6–7 mm) to reduce cell leakage. The stomach was then relocated to the abdominal cavity after the needle withdrawal, and the abdominal wall and skin were closed with 4–0 absorbable sutures.

We then compared different injection volumes (20 μL, 40 μL, and 60 μL) using MKN-45 cells suspended in PBS with the addition of trypan blue for the convenient observation of the cell leakage. A 30 G needle was used for the injection, and obvious cells oozing from the injection site were observed for the 60 μL injection volume (Figure 2B). Of note, the entry sites of the injection (indicated by red arrows) were clearly away from the cell bulges upon the injection’s completion (indicated in blue) for the 20 μL and 40 μL injection volumes, indicating that injecting a volume of ≤40 μL is suitable for orthotopic implantation. The volume of 20 μL was used for the subsequent experiments.

We next sought to examine the effect of the needle size on the cell leakage. A total of 5 × 10^4^ luciferase-expressing MKN-45 cells in a 20 μL volume were inoculated using needles of different gauges (25 G, 26 G, 27 G, 29 G, and 30 G). In vivo and ex vivo bioluminescence imaging showed that needle gauges ≤27 G lead to a high risk of severe cell leakage. At 2 weeks after the orthotopic implantation, 3–4 mice in the 25 G, 26 G, and 27 G groups showed obvious cell leakage (Figure 2C), even with no tumor detected in the stomach in many cases (e.g., 25 G: #3 and #5; 26 G: #1; 27 G: #1–#3) (Figure 2D). Taken together, our results confirmed that the injection of tumor cell suspensions in PBS at low volumes (≤40 μL) using a 29 G or 30 G needle caused a minimal risk of cell leakage. Moreover, the removal of the Matrigel from our procedure further reduced the cost of this experiment.

### 3.3. Recapitulation of Tumor Growth, Invasion, and Metastasis by Orthotopic Implantation of MKN-45 Cells in Suspensions

**Figure 3:**
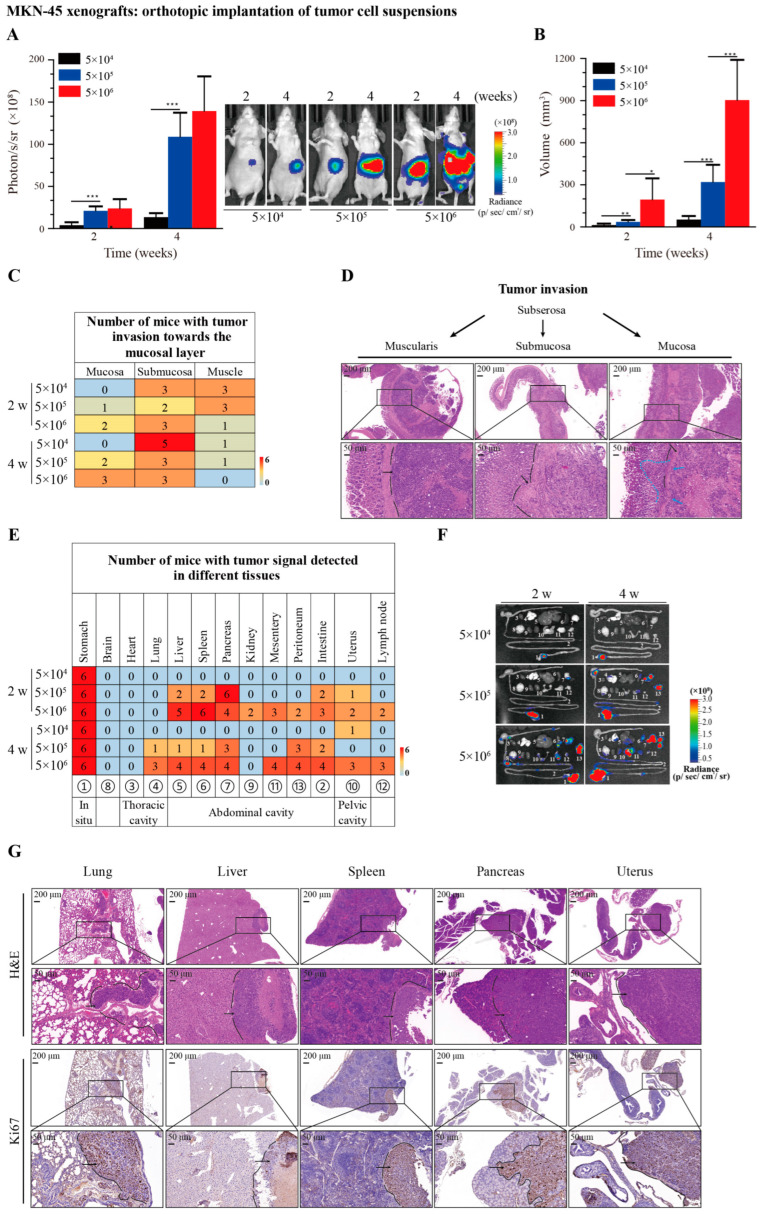
High engraftment and metastatic rates for orthotopic implantation of MKN-45 cells in suspensions. (**A**) In vivo monitoring of tumor growth and progression using bioluminescence imaging. Quantitative analysis of bioluminescence intensity (**left**) and representative images at 2 and 4 weeks (**right**) after orthotopic implantation of indicated numbers of MKN-45 cells suspended in 20 µL of PBS using 30 G needle. (**B**) Primary tumor volumes at 2 and 4 weeks after orthotopic implantation. (**C**) Degree of tumor invasion determined using H&E staining across the transverse section of the gastric wall. Summary of number of mice with tumor invasion toward the mucosal layer. (**D**) Representative images of tumor invasion toward the mucosal layer. The black lines and arrows indicate the boundary and region of the tumor mass that invaded different layers of stomach tissue. Blue arrows indicate the breakthrough loci of tumor cells from the submucosa to the mucosa. Blue dotted line indicates tumor mass that invaded the layer of the mucosa. (**E**) Summary of tumor metastasis. Summary of number of mice with tumor signal detected in different tissues and organs. (**F**) Representative images of ex vivo bioluminescence imaging. The following tissues and organs are shown: 1. stomach, 2. intestine, 3. heart, 4. lung, 5. liver, 6. spleen, 7. pancreas, 8. brain, 9. kidney, 10. uterus, 11. mesentery, 12. lymph nodes from axilla and inguinal areas, and 13. peritoneum. (**G**) Representative images of H&E and Ki67 stainings, detecting tumor metastasis in indicated organs. Representative metastatic loci in liver (5 × 10^6^ cohort at 2 weeks), spleen (5 × 10^6^ cohort at 2 weeks), uterus (5 × 10^6^ cohort at 2 weeks), pancreas (5 × 10^5^ cohort at 2 weeks), and lung (5 × 10^5^ cohort at 4 weeks). Data represent the mean ± SEM of 6 mice in each group. * *p* < 0.05; ** *p* < 0.01, *** *p* < 0.001.

Having established the procedure for generating the orthotopic GC xenograft model using cell suspensions, we next examined the engraftment and metastatic rates of this model in detail. To do this, we set up three groups by inoculating 5 × 10^4^, 5 × 10^5^, or 5 × 10^6^ MKN-45 cells suspended in 20 μL of PBS in the sub-serosal area of the gastric corpus/antrum junction with a 30 G needle. The mice were examined at week 2 and week 4 post implantation. The quantification of the bioluminescence intensity and measurement of the tumor size showed both dose- and time-dependent tumor growth and progression (Figure 3A,B). Accordingly, both the dose- and time-dependent progressions of tumor invasion were nicely recapitulated in these mice (Figure 3C,D). The metastatic rate increased with increasing number of inoculated cells, but metastasis was barely detected in the 5 × 10^4^ cohort by week 4 (Figure 3E,F). This is in good agreement with the model for transplanting tumor fragments, as a 1 mm^3^ tumor fragment roughly contains 1–5 × 10^4^ cells (Figure 1F,G). The analysis of the tumor metastasis showed most metastasis occurred in the peritoneal and pelvic cavities. Notably, lung metastasis was observed in the 5 × 10^5^ and 5 × 10^6^ cohorts, with 1 and 3 out of 6 mice detected by week 4, respectively (Figure 3E,F). In addition, lymph node metastasis was also detected in the 5 × 10^6^ cohort. Tumor metastasis in some of these major organs was further confirmed by H&E and Ki67 staining (Figure 3G). These results confirmed that the orthotopic implantation of MKN-45 cell suspensions can reliably recapitulate tumor growth, invasion, and metastasis.

**Figure 4:**
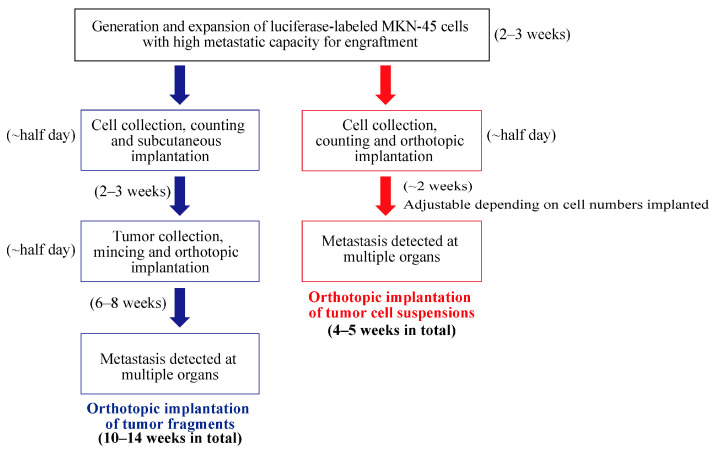
Summary of the experimental timelines to establish MKN-45 orthotopic xenografts using these two methods.

Moreover, compared with the implantation of tumor fragments, the implantation of tumor cell suspensions allows for a more flexible adjustment of the number of tumor cells implanted to control the rates of tumor progression and metastasis. The experimental timelines to establish MKN-45 orthotopic xenografts using these two methods are summarized in Figure 4. Our procedure significantly shortens the experimental timeline, reducing it to 4–5 weeks from 10–12 weeks required for engrafting tumor fragments.

### 3.4. High Engraftment Rates in 23132/87 and HGC-27 Orthotopic Xenograft Models via Direct Implantation of Tumor Cell Suspensions

**Figure 5:**
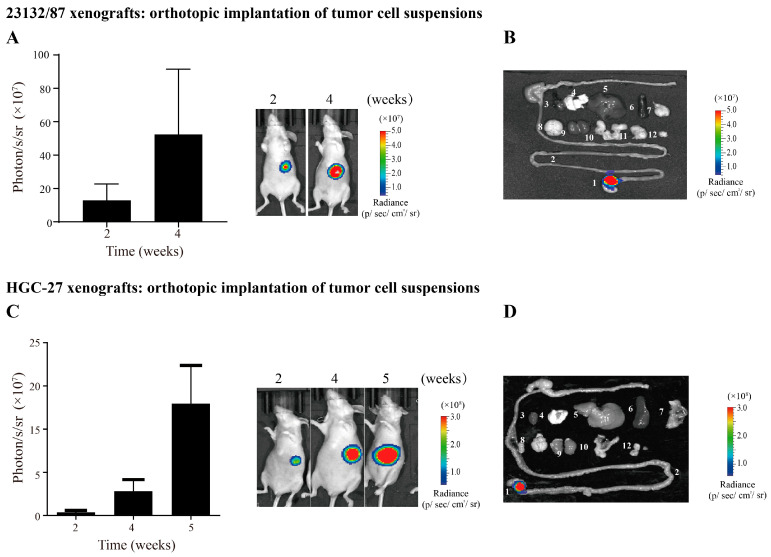
High engraftment rates and no cell leakage achieved by orthotopic implantation of 23132/87 and HGC-27 cells in suspensions. (**A**) In vivo bioluminescence imaging at 2 and 4 weeks after orthotopic implantation of 5 × 10^6^ luciferase-expressing 23132/87 cells suspended in 20 µL of PBS using 30 G needle. Four mice were used in each group. (**B**) Ex vivo bioluminescence imaging of major organs and tissues collected from the above mice at 4 weeks post implantation. The following tissues and organs are shown: 1. stomach, 2. intestine, 3. heart, 4. lung, 5. liver, 6. spleen, 7. pancreas, 8. brain, 9. kidney, 10. uterus, 11. mesentery, and 12. lymph nodes from axilla and inguinal areas. (**C**,**D**) Experiments similar to those in (**A**,**B**) were performed with HGC-27 cells. Data represent the mean ± SEM of 3–4 mice in each group.

To further confirm the high engraftment rates and low risk of leakage of our model, we set up another two orthotopic xenograft models by the direct implantation of tumor cell suspensions: 23132/87 was established from the primary tumor, while HGC-27 was established from the metastatic lymph node of gastric cancer [24,25]. Similarly, 5 × 10^6^ luciferase-expressing 23132/87 cells suspended in 20 μL of PBS were inoculated in the sub-serosal area of the gastric corpus/antrum junction with a 30 G needle. The mice were examined at week 2 and week 4 post implantation. The quantification of the bioluminescence intensity showed time-dependent tumor growth and progression (Figure 5A). Consistent with the weak metastatic ability of the 23132/87 cells, ex vivo bioluminescence imaging showed that all the mice formed tumors in the stomach, with no tumor signals detected in other organs (Figure 5B). Although a previous study used tail vein injections to mimic the lung metastasis of HGC-27 [26], the orthotopic implantation of HGC-27 cells showed no metastasis by week 5 (Figure 5C,D). These results further confirmed the high engraftment rates and minimal risk of cell leakage in our model system.

## 4. Discussion

The implantation of intact tumor fragments is commonly used to develop orthotopic xenograft models of gastric cancer at high success rates [9,10]. In recent years, the orthotopic implantation of tumor cell suspensions using different techniques has also been reported and has shown various engraftment and metastatic rates [12,13,14]. To date, no parallel studies have been conducted to directly compare the engraftment and metastatic rates of tumor fragments versus tumor cell suspensions from cultures. Although the study by Furukawa et al. in 1993 showed that the engraftment and metastatic rates for engrafting cell suspensions were 50% and 0% compared with 100% and 70%, respectively, for implanting intact tumor fragments, it should be noted that the cell suspensions used for the orthotopic implantation in this study were obtained by the enzymatic digestion of subcutaneous tumors, which may have compromised the engraftment and metastatic capabilities of the tumor cells [15]. Therefore, there is no conclusive evidence indicating that tissue fragments are superior to tumor cell suspensions in terms of successful engraftment and metastatic spread.

In the present study, we conducted parallel investigations and demonstrated comparable high engraftment and metastatic rates between the implantations of tumor fragments and cell suspensions in MKN-45 cells with a strong metastatic ability. Importantly, compared with the orthotopic implantation of tumor fragments, our approach significantly shortens the experimental timeline, reducing it to 4–5 weeks from 10–12 weeks required for engrafting tumor fragments. Moreover, our approach allows for the flexible adjustment of the number of tumor cells implanted and, thus, the rates of tumor progression and metastasis. As we generated the orthotopic xenografts by directly implanting tumor cells collected from cultures into mice without multiple rounds of engraftment, we suspect that the orthotopic xenografts should retain the heterogeneity of the original tumor cell lines quite well. In the case of the primary samples, the use of multi-regional sampling and subsequent implantation can improve the maintenance of tumor heterogeneity. From this aspect, we suspect that the implantation of a well-mixed tumor suspension may better preserve tumor heterogeneity, which may, however, come at the cost of compromising the tumor microenvironment [27].

Our model successfully recapitulated both time- and dose-dependent progressions of tumor invasion and metastasis. Importantly, rapid tumor growth and metastasis can be achieved by implanting higher numbers of tumor cells, with evident metastasis occurring within ~2 weeks when implanting 5 × 10^5^ and 5 × 10^6^ cells. Although increasing the size of the tumor fragments may reduce the time required for metastasis, it is not likely to achieve similar rates owing to the low number of tumor cells in each fragment (1–5 × 10^4^ cells in a 1 mm^3^ tumor fragment). Moreover, larger tumor fragments carry an increased risk of central necrosis and ischemia due to inadequate blood supply [28], which may conversely lead to lower rates of engraftment and growth. In GC patients, metastatic progression occurs through multiple routes, including lymphatic, hematogenous, peritoneal, as well as ovarian routes [29], all of which were present in the MKN-45 orthotopic models. Thus, our results demonstrated that the orthotopic implantation of tumor cell suspensions can reliably recapitulate tumor growth, invasion, and metastasis, for which all the rates are adjustable by implanting different numbers of tumor cells.

Unexpected cell leakage into the abdominal cavity is one of the major obstacles that limits the widespread use of the direct orthotopic implantation of tumor cell suspensions. We suspect that suboptimal surgical procedures and the use of inappropriate needle sizes or sample volumes could contribute to an increased risk of cell leakage. Previous studies have reported the use of needles with various diameters, including 30 G [19,30], 29 G [18], and 26 G [31], for the orthotopic injection of tumor cells, while some studies did not specify the needle size that was used [12]. In our study, we systematically compared the effects of different injection volumes and needle sizes on cell leakage. We demonstrated that injecting cells at low volumes (≤40 μL) using 29 G or 30 G needles at a sufficient distance of 6–7 mm between the entry loading sites minimized the risk of cell leakage. Moreover, our procedure for injecting tumor cell suspensions without the addition of Matrigel not only reduces costs but also eliminates the unpredictable effects of Matrigel on tumor growth and metastasis.

However, there are several limitations to our model system, which should be addressed in future studies. First, human gastric cancer primarily originates from the innermost mucosal layer and penetrates outward into the submucosa, muscularis proper, or serosa [32]. In our study, intact tumor fragments or cells in suspensions were implanted in the sub-serosal area of the stomach, which allows for both inward and outward tumor invasion. However, owing to the very thin gastric wall in mice (approximately 0.5 mm for BALB/c nude mice), the precise inoculation of tumor cells in the mucosal layer from the outside of the stomach is technically challenging. Endoscopy-guided implantation may potentially overcome this issue. Second, we did not evaluate the engraftment and metastatic rates of primary GC samples in cell suspensions (e.g., ex vivo cultured patient-derived organoids) in the current study. Third, we conducted a whole-organ ex vivo study. Owing to the intense signal of the primary lesion, sometimes weak signals are obscured. If we conduct an ex vivo study of the primary and other lesions separately, more metastases may be detected.

## 5. Conclusions

In conclusion, we provided a highly reproducible procedure with a shortened experimental timeline, low cost, and minimized risk of cell leakage for establishing orthotopic GC xenografts via the direct implantation of tumor cell suspensions. Compared with the routine method to establish orthotopic xenograft models by engrafting intact tumor fragments, our approach significantly shortens the experimental timeline and allows for the flexible adjustment of the number of tumor cells implanted to control the rate of tumor progression. Thus, our work offers valuable tools for expanding the application range of orthotopic models in gastric cancer research.

## Data Availability

All the data supporting this study are available from the corresponding author upon reasonable request.
